# Some Practical Remarks upon the Subject of Insanity*Read before the Alumni of the Buffalo Medical College.

**Published:** 1883-04

**Authors:** Judson B. Andrews

**Affiliations:** Superintendent State Asylum for the Insane, Buffalo, N. Y.


BUFFALO

### Medical and Surgical Journal.

Vol. XXII. APRIL, 1883. No. 9.

### Original Communications.

### Some Practical Remarks upon the Subject of Insanity.*

By Judson B. Andrews, M. D.

Superintendent State Asylum for the Insane, Buffalo, N. Y.

Gentlemen—I now address you at the request of Dr. Barrett, the Chairman of your Executive Committee. By his designation of the subject, I am relieved of one of the difficulties of the situation. He chose for the occasion, "Some practical remarks upon the subject of insanity." These remarks have been written amid the press of other duties, and hence I ask your indulgence.

Insanity is constantly attracting more attention on the part of the medical profession and of the general public. The physician is interested in it, as a medical subject, while the public consider most largely its humanitarian and economic aspects. It has become an important element in social science, which deals with the questions of its prevention, causation, and the best methods of care of the insane, consistent with public policy and the duty of society toward the sick and afflicted. It is not this

*Read before the Alumni of the Buffalo Medical College.

view of the subject on which I wish to speak to you, since, as physicians, you are more directly interested in the medical side.

The first question that confronts us is: What is insanity? This is answered in the most general way by the single word disease, or as some more critical would say, a symptom of disease. It would be differently limited and qualified according to the theory entertained by the speaker. By one it would be called "disease of the mind," by another, "disease of the brain whose secretion is mind," and by a third, "abnormal mental action depending on disease of the brain."

In the first answer we recognize the metaphysical view or the spiritual theory which for ages controlled not only medical thought but the intelligence of the times. This theory recognized insanity as a perversion of the soul or the possession of an evil spirit, the punishment inflicted by a divine power for sins committed.

Hence the belief in demonology, and from this divine control, the belief in the inspiration of the lunatic, who came to be consulted as a soothsayer, a foreteller of future events; and the incoherence and raving of the mad man were oracular responses to those who sought advice and guidance in their affairs. But we need not go back to the remote ages to find evidences of this theory; it still exercises its influence; we have it constantly before us in the nomenclature of insanity, in the terms mental disease, disease of the mind, and a common idea that in the treatment of insanity there are some peculiar medicines employed to produce an effect on the mind. This theory would make insanity a sin to be cured by repentance and divine forgiveness, and for treatment the aid of the priest and not of the physician should be invoked. It is not infrequent, as you know, that the solace and prayers of the clergy are sought in cases of melancholia laboring under religious delusions. This is certainly consistent with the idea that insanity is a disease of the mind.

Let us hear what one of the prominent advocates of this theory has to say: "Insanity is a loss of moral liberty. It never depends upon a physical cause. It is not a disease of the body but of the mind—a sin. It is not and cannot be hereditary because the thinking ego, the soul, is not hereditary. The man who has during his whole life before his eyes and in his heart the image of God, has no reason to fear that he will ever lose his reason. Man has a certain moral, which cannot be conquered by any physical power and which falls under the weight of his own faults."*

Under this theory the physician has nothing to do, as without physical disease there is no call for his advice or his drugs.

The logical outcome of this view does not offer any encouragement to the *spiritual adviser*, as the very existence of *disease* of the mind implies the possibility of its decay and death, and thus destroys the hope of immortality and future happiness.

The second theory of insanity, as a disease of the brain, whose function is mind, makes the mind simply a secretion from the brain. Nothing more than a force developed by the action of the brain. It is illustrated as follows: "As a good liver secretes good bile, a good candle gives good light, good coal a good fire, so does a good brain give good mind. When the brain is quiescent there is no mind." This theory, prominently brought forth a century ago by Cabanis, has at this time some advocates. It is the strictly materialistic view, and finds no place for the existence of the mind separate from the body. When vital action ceases and the organism dies mind is extinguished. The death of the body ends all. There is no future accountability, for man is bounded by his physical environment. Such a theory, although repugnant to the Christian world, recognizes the disease of the brain as the essential factor which calls for medical care and treatment.

The third and most commonly accepted theory of insanity assumes the mind as having a separate and independent exist-

---

*Heinroth, a celebrated German Psychologist.

ence apart from the body, and that the brain is the organ of the mind. It views the mind as the spiritual part of man, the agent which uses the material organ, the brain, as the instrument of communication with the external world. It is the ego of the metaphysician, the reasoning soul of the theologian, eternal, immortal, indivisible, subject neither to disease nor decay. It is separated from the body, though it maintains a relation to it so intimate as hitherto to have eluded the profoundest research to explain.

In accord with these theories, when the brain is in a state of health the mind acts normally, unimpeded and unfettered in its operations. This is a normal mental state, or sanity.

In further explanation the materialist would say, that in a healthy state of the brain, the secretion, or emanation, or mind must be normal, and therefore sanity exists.

By the accepted theory, the brain, affected by disease, gives evidence of the change by exaggerated, diminished or perverted mental action, which is insanity.

It is not necessary for us to attempt to prove that insanity depends upon disease of the brain. The proof is constantly before us, and the researches of chemistry and of the microscope in the laboratory of the pathologist are constantly adding to them. Though it may be claimed by cavilers that in some instances insanity has existed, and no post mortem lesions have been discovered, it is only fair to assume, from the great mass of evidence, that they do exist, but are not detected, either from lack of thoroughness in the examinations or because they are of a character which elude our present means of investigation.

In reply to the question, what is insanity, you will expect from me a more definite answer than has been given. To define it so as to include all its phases and to exclude all such mental states as are not properly classed under the term is not an easy task. To attempt such a strict definition would only lead to failure.

Burton, in his "Anatomy of Melancholy," says: "If you would describe melancholy, describe a phantastical conceit, a corrupt imagination, vain thoughts and different, which, who can do? The four and twenty letters make no more variety of words in diverse languages than melancholy conceits produce diversity of symptoms in several persons."

The truth of this will be at once appreciated if we consider the infinite variety of morbid mental phenomena, which it is proposed to include within the limits of a definition.

Many definitions have been presented, some of which in their frequent repetition are familiar to all. Locke defines insanity by the pithy sentence: "Madmen reason correctly, but from wrong premises." This, if true, could apply to a small part, only, of the insane, and is therefore useless as a general statement.

Cullen says it is "a lesion of the intellectual faculties without pyrexia or coma." This is a negative, so far as disease is concerned, and is not sufficiently comprehensive. Combe says, "Insanity is a morbid action in one, in several, or in the whole of the cerebral organs, and as its necessary consequence, functional derangement in one, or in several, or in the whole of the mental faculties." This is a phrenological definition and divides the brain into organs, and the mind into separate faculties, a theory which has not stood the test of time and experience.

We might continue these quotations to a tiresome length, but forbear. We present a definition which more nearly meets the requirements, and while it is not claimed to be beyond the reach of criticism, it has the sanction of high authority and is believed to fulfill the requirements more nearly than any of which we have knowledge. "Insanity is a more or less prolonged departure in the individual from his normal mode of thinking, feeling or acting, the result of disease of the brain."

This comprises the whole of man's mental life, as manifested in the emotions, intellect and will, and places the disturbance of any or all parts of it, which amounts to insanity, upon the firm basis of disease, disease of the brain, the only basis which is tenable ground for the physician. It will be noticed, and this is a most important part of the definition, that the individual is to be compared with himself. To judge of insanity there is no fixed criterion, no definite standard by which the emotions, thoughts or conduct of the individual are to be measured, and those which come up to the mark are to be set aside as indicating sanity, while those which vary from it with equal certainty denote the presence of insanity. As you know and act upon the knowledge in your daily experience, every person is individualized and fully identified by his physical and mental characteristics. These relate to the features, to the form, gait and movements of the body, to the modes of thought and expression, to certain manifestations of emotional life, and to a standard of morality which controls the conduct. All of these characteristics, as modified by the circumstances of birth, education, occupation, social position and the like conditions, constitute the personality, the norm of the individual. Recognizing the principle that every person is a law unto himself, it is necessary, before we predicate insanity in any given case, to become familiar with the previous history of the individual and note the departure from the normal state.

Another important feature of the definition is in the words a more or less prolonged departure. This is intended to exclude cases of temporary delirium from fever, from injuries, from the action of narcotic drugs, alcohol, etc. Although any of these may be the efficient cause of insanity and actually produce it, yet, as ordinarily met with in practice, such changes do not amount to insanity.

Such then are the changes indicative of insanity. To repeat, they must constitute a departure in the individual from his normal mode of feeling, thinking or acting; the departure must be more or less prolonged and not of temporary duration, and essentially important, they must be the offspring of disease of the brain.

Dr. Hack Tuke, a most competent observer, speaking of this subject, uses the following language: "Seeing, in short, that our mental powers depend in this life upon the health and integrity of a vast number of brain cells; that this health and integrity require suitable nutrition, both as to quantity and quality, and a free supply of oxygen, it must be that the mind, in its contact with the world, will frequently suffer; indeed the wonder is, not that so many become insane in the midst of so great a neglect of the laws of nature, so much vice, lamentable want of mental cultivation on the one hand and excessive unregulated mental work on the other, but that so many escape." In this sentence are crowded the principal factors for the physiological action of the brain, as well as many of the causes of mental disturbance, stated in plain, familiar words.

We now direct your attention to some of the first symptoms of mental disturbance. They have a peculiar significance to you as physicians, and their early recognition is of great moment to the patient, in enabling you to adopt such measures as may avert a threatened attack of insanity. The pathologic processes in the brain are preceded by certain changes in the physical system and in the mental habits of the individual, which, if rightly understood and properly met, may yield to medical treatment.

These symptoms will be found in one or more of the following directions: First, disturbance of function; second, of sensation, and third, of feeling. Of the first, impairment or loss of sleep is the most common. Of the necessity of the integrity of this function for the health of the body and for the proper exercise of the mental powers, and of the serious consequences of its continued disturbance, I need not speak. You know the story too well. You have painfully watched the exhausting effect upon your patient in the failure of health, and loss of strength, and seen the mind pervaded with feverish restlessness, giving way to groundless suspicions and fears, till after a vain struggle it yields to the malign influence, and insanity is fully established. In a normal, healthy condition sleep naturally follows activity, action and repose being the physiological sequence. In disordered states of the brain the operation of this law is often interrupted; action is but the stimulus or spur to increased activity. From the disturbance of the circulation within the cranium, there results an irritability of nerve tissue which may prolong the existing excitement.

Disturbance of other functions frequently exist; among them are loss of appetite, indigestion, constipation and general derangement of the secretions. The circulation is affected, the heart showing signs of feebleness, and the pulse increased in frequency and diminished in force. The vaso-motor disturbance, marked by suffusion of the neck and face, indicates depression and irregularity of nerve action.

On the other hand, the heart's action may be increased, the pulse full and bounding, the face flushed, the eyes injected and the pupils contracted, evidences of a hyperæmic or congested state of cerebral vessels. Again, there may be palor, with dilated pupil and coldness of the extremities, indicative of anæmia and a falling off in the quality of the blood.

With the conditions of the circulatory system, and depending upon them, there may be changes in the sensations.

The most frequent relate to the head. There may be headache, or pain, and a sense of weight, where an apparent effort is required to hold it up, or of lightness, as if the skull was empty. The patient sometimes compares the sensation to a peculiar feeling, as if the head was made of wood; occasionally these form the basis of delusion. A very common complaint is made of the sensation of a band drawn tightly about the head, of heat in the vertex, of throbbing in the arteries, and of singing in the ears. Other parts of the body are also affected. There are abnormal sensations referred to the abdominal organs, especially in hypochondriacal cases which develop into fixed delusions.

Changes on the side of the feelings next attract our attention and complete the array of symptoms strongly indicative of the approaching attack of insanity. "Among the emotional disturbances may be enumerated slight depression of spirits, especially if this alternates with a sense of exaltation and buoyancy." "If the world seems unreal, if the path of life seems gloomy, if the slightest circumstances bring tears to the eyes, these are incipient symptoms of irregular brain action." "So irritability of temper, moroseness and irascibility in the naturally sweet and amiable, suspiciousness in an open, trusting nature," change of manner toward those previously loved, unfounded jealousies in the beginning vague and shadowy, a feeling of the existence of opposition to the individual and to all his plans, forebodings of impending evil indefinite and indefinable, indecision of purpose, not only in regard to matters of importance, but in trivial affairs of life, as of the clothing to be put on, the food to be eaten, all of these point in the same direction.

An unnatural levity in contrast to former disposition, increased mental activity, leading the quiet and reserved to become talkative, obtrusive, self-confident and boastful, in short, changes in the habits, conduct and feelings of the individual without adequate cause, when combined with the physical changes before mentioned, may well excite question and lead to inquiry as to the mental state of the person concerned.

There are cases of insanity more rapid and open in their occurrence, such as follow acute attacks of disease, injuries, excessive use of stimulants, and the like, which present the more marked symptoms, either the noise and violence of mania or such a degree of depression as leaves no question in diagnosis, or of the necessity of immediate treatment. We have, however, chosen to speak of those forms which give warning of their approach and thus enable the physician to put in practice the knowledge of his art in the direction of prevention.

It is the duty of the profession to recognize the significance of these changes and to take the necessary steps to guard against their further progress toward insanity.

Success will not always crown our efforts, but they should not be intermitted. The public look to the profession for relief from the oppressive burden of lunacy with which it has to contend and for which it has to care. Much has been written regarding the prevention of insanity, but the people do not heed the warning voice. Led by the desire to excel in the strife for riches, position and power, or, on the other hand, compelled to work far beyond their strength, to struggle with poverty and actual want, they press forward, or are forced on by the circumstances of life till some additional burden, in the form of grief anxiety, or ill health, develops the changes indicative of mental disturbance. It may not be out of place for me to ask if we do not too often under-value the importance of these symptoms, or misinterpret them to the loss of valuable time and to the detriment of our patients. Do we not too often delay to institute proper measures in the way of advice and treatment till the disease is openly declared and the most favorable time for benefit is past? It is too late in the day for me to attempt to prove to you the already established axiom that the early stage of insanity is the curable one, and that in the years after, treatment is mostly barren of recoveries.

In the diagnosis of insanity we may be materially aided, if we bear in mind that most frequently the disease has its history, in the changes brought to your notice, and that in the greater number of cases there is depression of spirits, varying in degree from slight despondency to well marked melancholy. At this stage it may be impossible for us to say what may be the result, whether we will have to treat a case of melancholia, or whether, after a period of longer or shorter duration, the symptoms of mania may not supervene.

Continuing the consideration of the subject, we come now to speak of cases of fully developed insanity, and I venture, at this point, to give some hints of the methods of dealing with them which experience has shown to be valuable; and first, as regards the examination of the patient whose mental state is in question.

Gather from the friends all the information they may have to give relating to the past and present history and normal characteristics of your patient, and especially all the facts which tend to induce a belief in the existence of mental disorder. Then seek an interview with the patient, either alone or with a person who has the closest friendship and the fullest confidence combined with the most intimate knowledge of the case. Exert yourself to gain the confidence of the individual to be examined. This can often be accomplished by the introduction of some subject in which he is interested, perhaps his occupation, his surroundings, or some topic upon which you know he has gained, by study or experience, a special degree of knowledge. This can be easily turned to the history of the individual, the state of his health, when all the organs of the body can be examined, and the influences of a depressing or deteriorating character can be discovered. Follow up the course thus begun; make it to include the habits, social relations, religious views, the occupation or business affairs, so far as they may be a source of anxiety and worry; in short, have before you the prominent and controlling factors in the physical, moral and mental condition, and note the departure from the normal state of the individual. Thus you will discover the delusions, if any exist, and be able to arrive at an intelligent opinion. If, however, you cannot satisfy yourself upon one visit, repeat it until you are satisfied you have arrived at the truth. You will be surprised at the readiness with which your questions are answered and trust is reposed, and still more of the appreciation which the patient has of his own condition.

Suppose he has melancholia with its depressing ideas, it is always well to inquire as to the presence of suicidal tendencies; put the question directly, though guardedly, to your patient, and you will usually be answered truthfully, and perhaps, to the astonishment of friends, he will narrate some attempt or preparation therefor, and thus put you on the watch against the success of any efforts in this direction. If such feelings exist, it is well to explain them as symptoms of the disease, which may be expected and which must be struggled against. It often fortifies the mind of the sufferer if the act is properly characterized as cowardly and sinful, and the terrible consequence to one's family and friends are pointed out. Such arguments sometimes furnish a moral support to the patient which will enable him to successfully combat this sad phase of his disease.

If delusions are expressed, it is well to characterize them as such and to expose their error. In case they are fixed and control the patient, neither argument nor persuasion will avail to remove them. Combating them vehemently only irritates, and strengthens the belief in their reality by the attempt of the patient to sustain them. Ridicule has no better effect. A calm and quiet denial, with a full statement of the facts, is true kindness to the patient, but if the state of mind is such that this induces disturbance or violence, it is better to pass it over in silence. Do not be led into an altercation, as this will destroy your influence. A mild and quiet manner in conversation, unruffled by any word or act of the patient, exercises a wonderfully restraining and controlling influence.

Honest dealing with the insane gains their respect and confidence. If physicians and friends could only realize the harm and irritation produced by deception, it would never be resorted to. For a physician to gain access to a patient, to examine him for lunacy under an assumed name, or to conduct the examination under the plea of transacting some business, such as purchasing some of his supposed holdings, or selling property to the man, rich only in his imagination, is simply perpetrating a fraud which is rarely forgotten or forgiven by the patient. So, for friends to convey an insane person to an asylum under the plea of a visit to the institution, or that the asylum is a hotel where they can obtain a meal or stay for a day while transacting business, only exposes them to his anger when the deception is discovered, and undermines his faith in mankind. It is an injustice and does injury to the patient.

There is quite a common belief, with which you are, no doubt, familiar, that the insane are not to be opposed, but humored in their delusions and peculiarities. In receiving the history of a case from friends, they frequently state—as if they desired credit for it and had done just the right thing—that the patient has had his own way and not been opposed in anything. He may have been allowed to turn night into day, and day into night, to go wherever and whenever he chose, to make purchases, to dispose of property, to follow his vagaries of dress and conduct, in short, all who come in contact with him have yielded their good sense and judgment to the whims of the insane man under the mistaken idea that it is a duty they owe the patient and the correct thing to do. It is not an act of kindness to assent to the delusions of the patient or be controlled by them. As a case in point a patient gives as proof of the truth of his claim that he is Jesus Christ, that his wife and family acknowledge the fact by kneeling to him and addressing him by that title. Another patient's delusions are the more fully established, because some injudicious friends imposed upon his credulity by making imaginary sales and drawing bogus deeds and contracts, till his delusive wealth exceeds the real wealth of a Vanderbilt. These men were wronged and injured by the conduct of their friends, as their delusions are only the more firmly fixed.

The patients whose delusions of greatness and power lead them to believe they are kings and queens, or hold other high positions of honor, if they are addressed as such, treated with the mock respect due their supposed rank, and allowed to decorate their person, to confer titles upon others—they are only strengthened in their belief, and when they recover will know they have been made the butt for ridicule, and that their suffering has been but the jest of others' folly. The insane should be treated as intelligent and responsible beings, and led to conform so far as may be possible to the ordinary regularity of life and habits. In this way you will do them the most good and preserve your own self-respect.

The changed conditions, induced by insanity, often demand the removal of the patient from the surroundings in which they are generated and fostered. In some cases, especially of mild melancholia, without fixed delusive ideas, a visit to the house of a friend, or the change of scene, gained by traveling with a judicious and cheerful companion, may be of benefit. This should not, however, be recommended, unless the patient is in a physical state not only to endure the fatigue and inconveniences of a journey without injury, but he must be in a mental state to be diverted, and to have his attention attracted by the changing views and scenery. It is cruel to make a sick and feeble patient, who is so absorbed in his unhappy state as not to appreciate the constantly varying surroundings, leave the comforts and society of home for the discomforts of life in a hotel and the weariness of travel. In such a case irreparable injury may be done to the patient. Whenever this step is proposed, it should receive the careful consideration of a physician before it is recommended. It is not a form of treatment which can safely be adopted in a routine way.

In most cases of insanity removal to a hospital, adapted to the care of the insane, offers the best advantages to the patient, with the greatest protection to the public, and upon the physician the law places the responsibility of deciding upon the propriety of this course. It requires the certificate of two physicians, duly qualified as examiners in lunacy, to be approved by a judge of the district in which the patient resides. Under this law many thousand patients have been committed to the asylums of the State, and the judgment of the physicians has been almost universally approved by the judiciary, so that the right of a trial by jury, which is made optional with the judge, has rarely been exercised. This work has not, on the part, either of the medical or legal profession, been a matter of routine or careless indifference, but has been carefully and conscientiously performed.

The medical profession is not justly open to the charge so flippantly made and so often reiterated, by those whose ignorance or prejudice is wonderfully exhibited when they come to speak of lunacy matters, of conniving, for substantial reasons, at committing patients to an asylum in disregard of the rights of the individual. There is no basis or shadow of truth for any such statement, as experience shows that the profession is as cautious in giving certificates of lunacy as in the duties which involve the life of the individual, and that it acts from the same high motives, the good of the patient. This very cautiousness sometimes, however, works an injury to the patient, as steps for his care are put off till some act of violence, toward himself or others, makes action a necessity, when it is found that the disease has acquired a foothold which destroys all reasonable hope of recovery.

This is owing in part to the sentiment of the community, the influence of which cannot have escaped your notice. In other forms of disease the judgment of the physician is ordinarily accepted without question, but in insanity, a form of disease which presents special difficulties in its diagnosis, there is a freedom of non-professional opinion which assumes to question, not only the existence of the disease, but the motives of the physicians and friends in adopting a course of treatment sanctioned by law and acknowledged to furnish the best chances of recovery to the patient. This opinion is often based upon the delusions of the insane. Unfounded jealousies and assertions of conspiracies to gain possession of the property of the patient, and the like delusions, are accepted as realities and create a feeling as senseless as it is unjust.

There is a tendency to consider the question of a man's insanity in the light of a political matter, to be decided by a majority vote, and that every one in the community is entitled to that vote. It is on this principle that a trial by jury is demanded, as if a jury of six or more men were required to tell whether a man was sick and needed treatment in a hospital. In reading over the proposed legislation, on the subject of insanity, one might fairly believe that a sick and insane man was somehow mistaken for a criminal. We find, in many of the proposed changes, the following language, that the "accused" "shall have a trial," "before a jury" and "be defended by counsel." What has the poor man been guilty of that all the paraphernalia of the criminal court should be brought into use. We have always supposed that the insane were sick and disordered people who needed the care of a physician, and that, as in other form of disease, he was the proper person to diagnosticate the case and prescribe the treatment. This demand for a trial by jury is the one radical change in the law of commitment which seems, at the present time, to fill the public mind. This law has been in operation for several years in the State of Illinois, and it may not be out of place for me to quote the opinion of a most competent observer as to its practical results. Dr. Carriel, the Superintendent of the Central Illinois Asylum, in his last report, uses the following language: "It is probably in the experience of every superintendent of the State, as it certainly has been in mine, to notice how many have been deterred from making application for their friend's admission to a hospital on account of the laws of this State requiring a public trial, a judge, jury and lawyers, just as in a criminal proceeding. I say many are kept out of the hospital in the early stage of disease, on account of the repugnance and dislike, in the minds of friends, to this public trial. Many such cases come under my personal notice. It seems strange that our law makers should be so tenacious in retaining the provision. If a man breaks his leg or is stricken down with fever, it is not deemed necessary to have a public examination and order for commitment before he can be placed in a hospital for proper treatment, but if a poor woman, from nervous shock in child bed, is seized with delirium and imagines that those whom she loved best are now turned against her, and have become her worst enemies, she must be taken in her weakness, through mud or over frozen roads, often many miles, to the county-seat, to have these things publicly told about her; and all this for the protection of liberties of the citizen, it is claimed, when in no other State is such a law found necessary, and although the charge that sane persons have been confined, for selfish or corrupt purposes, in any State hospital for the insane in this country, cannot be substantiated. We live in hope that even in this State the day will come when the liberties of her sick and afflicted may be thought safe in the hands of the medical profession, whose prerogative it should be to decide the question of the necessity or desirability of commitment to a hospital; then will one source of chronic insanity be removed."

This change of law from the present statute of commitment by certificate of two physicians, with the other safeguards specified, to a commitment only on trial by a jury, is demanded on the ground of greater safety to the rights of the citizens and to prevent sane people from being sent to asylums. Let us, for a moment, examine this phase of the subject. In a work written by Dr. Ordronaux, late Commissioner in Lunacy, entitled "Judicial Aspects of Insanity," and which is an acknowledged authority in the legal profession, we find the following remarks upon the subject of the trial of questions of mental state by juries:

"The experience of every day adds weight to the conviction that a jury of laymen is an unsafe tribunal to which to commit an issue of insanity. So far from such a tribunal affording any protection to personal liberty, it happens to operate so frequently in an opposite and oppressive direction that its findings have ceased to have any weight in the eyes of scientists. As an example of the perils to personal liberty from jury trials in issues of insanity, may be cited the report of the Superintendent of the State Asylum at Utica for the year 1872, which shows that fourteen cases were discharged as not insane." "All of these," Dr. Gray says, "were committed under public authority and on certificates of insanity, or a trial by jury. On the other hand, no single instance of error in diagnosis occurred either in public or private cases when the family physician has made the examination and recommended the case to be sent to the asylum." This was written before the law of 1874 was enacted, which embodied the feature of certificates of two medical men—approved by judicial sanction. The operation of this law has been highly satisfactory and needs no radical changes, either for the protection of society or of the individual.

Individual rights are fully protected by the clause which gives the judge discretionary power to "call a jury in each case to determine the question of lunacy;' and still further the law provides for an appeal, in the following language :

"If any lunatic, committed under the provisions of this article or any friend in his behalf, be dissatisfied with any final decision or order of a county judge, special county judge, etc., he may, within three days after such order or decision, appeal therefrom to a justice of the Supreme Court, who shall, thereupon, stay his being sent out of the county, and forthwith call a jury to decide upon the fact of lunacy."

Perhaps the best proof of the sufficiency of the law of commitment is found in the fact that these additional safeguards of trial by jury and appeal from the judge's approval are rarely, if ever, called into use. They stand upon the statute books as a reserve power of the law to meet any possible emergency, but which it has not been necessary to invoke.

Why, then, the demand for a trial by jury, in every case, when, although legally provided for, it is not used in any cases? It would seem that ignorance of the present statutes could alone give rise to the call for a change. There is one change we would advocate, and this is an addition to the present law. I refer to voluntary commitments to insane hospitals. Cases are occurring, from time to time, in which the individual appreciates his condition, sees insanity approaching, and desiring to place himself under proper care, seeks admission to an asylum. Here he is met by the legal provisions of two medical certificates, etc.

He cannot find any medical men who are willing to certify to his actual insanity, and the patient is obliged to wait till the disease has progressed to an advanced degree and the most hopeful period for recovery has passed. This is an injustice which can be prevented by making entrance to an asylum, like that to a general hospital, depend upon the desire of the sick person.

In conclusion, then, we repeat that after an extensive experience with the present law, no great changes are demanded, and that such as are needed should be in the direction of greater ease in gaining entrance to the asylums of the State.

